# Quantum yield and lifetime data analysis for the UV curable quantum dot nanocomposites

**DOI:** 10.1016/j.dib.2016.01.006

**Published:** 2016-01-13

**Authors:** Qi Cheng, Cui Liu, Wenjun Wei, Heng Xu, Qingliang You, Linling Zou, Xueqing Liu, Jiyan Liu, Yuan-Cheng Cao, Guang Zheng

**Affiliations:** a Key Laboratory of Optoelectronic Chemical Materials and Devices (Jianghan University), Ministry of Education, Wuhan 430056, China; b Flexible Display Materials and Technology Co-Innovation Centre of Hubei Province, Jianghan University, Wuhan 430056, China; c School of Physics and Information Engineering, Jianghan University, Wuhan 430056, China

## Abstract

The quantum yield (QY) and lifetime are the important parameters for the photoluminescent materials. The data here report the changes of the QY and lifetime for the quantum dot (QD) nanocomposite after the UV curing of the urethane acrylate prepolymer. The data were collected based on the water soluble CdTe QDs and urethane acrylate prepolymer. Colloidal QDs were in various concentration from 0.5×10^−3^ molL^−1^ to 10×10^−3^ molL^−1^, and 1% (wt%) 1173 was the photoinitiator. The QY before the curing was 56.3%, 57.8% and 58.6% for the QDs 510 nm, 540 nm and 620 nm, respectively. The QY after the curing was changed to 8.9%, 9.6% and 13.4% for the QDs 510 nm, 540 nm and 620 nm, respectively. Lifetime data showed that the lifetime was changed from 23.71 ns, 24.55 ns, 23.52 ns to 1.29 ns, 2.74 ns, 2.45 ns for the QDs 510 nm, 540 nm and 620 nm, respectively.

**Specifications Table**

**Table:** 

Subject area	*Physics, Chemistry*
More specific subject area	*Photoluminescent nanocomposite materials*
Type of data	*Table, text file, figure*
How data was acquired	*Combined Fluorescence lifetime and steady state Spectrometer, FLSP920*
Data format	*Raw, Analyzed*
Experimental factors	*QD colloidal was mixed with urethane acrylate prepolymer in various ratios.*
Experimental features	*UV irradiation was employed for* 5* s. Samples were directly applied to the Spectrometer for the requisition of data*
Data source location	*Wuhan, China PR*
Data accessibility	*Data is supplied with in this article*

**Value of the data**

• QY and lifetime are the important parameters for the photoluminescent devices to improve the efficiency [1], [2], [3], [4], [5], [6].
• The data are useful for the insights of the inactions between the nanoparticles and polymer matrix [7], [8], [9], [10], [11].
• The data are valuable for the material synthesis and devices design.

## 1 Data

Two figures and one table were provided to show the semiconductor CdTe QDs quantum yield and lifetime data before and after the polymerization of the urethane acrylate.

## 2 Experimental design, materials and methods

Fluorescent nanocrystal quantum dots (QDs) are used in various applications such as solar cell and optical-electronic devices [12], [13]. The quantum yield (QY) and lifetime are the important parameters for the material synthesis and device design. The data here report the changes of the quantum yield (QY) and lifetime after the UV curing of the urethane acrylate prepolymer.

The experiments were designed based on the water soluble CdTe QDs and hydrophilic prepolymer urethane acrylate; and the urethane acrylate can be polymerized under the UV irradiation.

### 2.1 CdTe QDs synthesis

The QDs were synthesized from the modified method according to the reference [1]. Briefly, 3-mercaptopropionic acid (MPA) was added into the CdCl_2_ solution in 50 mL of double distilled water in a round-bottomed flask and the pH was adjusted close to 10 in N_2_ bubbling. Then, the fresh-made NaHTe solution was applied to mix with the CdCl_2_ solution to reflux. Precipitation and centrifugation was applied to purify the QDs in water.

### 2.2 QD ink preparation

Colloidal QDs was mixed with urethane acrylate in various concentration from 0.5×10^−3^ molL^−1^ to 10×10^−3^ molL^−1^, and 1173 as the photoinitiator was at the concentration of 1% (wt%). The mixtures were sonicated 30 min before keeping still to remove the air bubbles. Then the QD ink was spread onto the glass slides for the UV irradiation.

### 2.3 UV curing

UV irradiation was carried out in the UV cabin with the 400 W light. The irradiation time was 5 s. Then the film was peeled off from the glass slide.

### 2.4 Data collections

For the QD inks, solution sample holder was applied to measure the QY and lifetime; for the film samples after the UV curing, solid sample holder was applied. For comparisons, samples before and after the UV curable were measured individually for the UY and lifetimes.

**Fig. 1:**
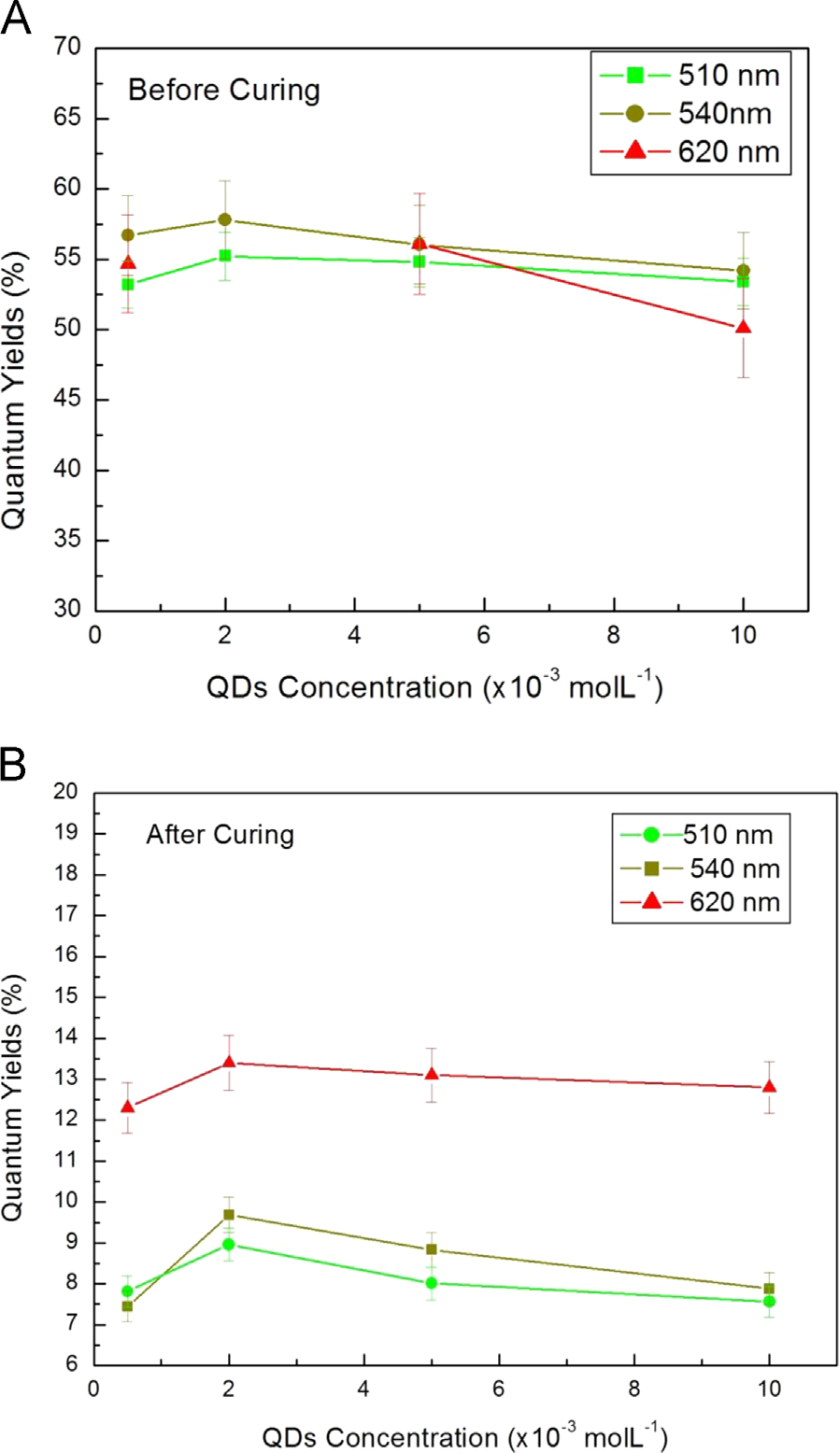
The QDs loadings effect on the QY of the nanocomposites.

Before the curing, the three type QDs all increased when the QDs concentration was increased from 0.5×10^−3^ molL^−1^ to 2×10^−3^ molL^−1^, then decreased when the QDs concentration increased. The QY before the curing was 56.3%, 57.8% and 58.6% for the QDs 510 nm, 540 nm and 620 nm, respectively (Fig. 1A). The QY after the curing was changed to 8.9%, 9.6% and 13.4% for the QDs 510 nm, 540 nm and 620 nm, respectively (Fig. 1B).

Lifetime data in Table 1 showed that the lifetime was changed from 23.71 ns, 24.55 ns, 23.52 ns to 1.29 ns, 2.74 ns, 2.45 ns for the QDs 510 nm, 540 nm and 620 nm, respectively.

## Figures and Tables

**Table 1:** The lifetime data.

λem (nm)	Before curing (ns)	After curing (ns)
510	23.71	1.29
540	24.55	2.74
620	23.52	2.45
