# Supplementary material for: Quantum yield and lifetime data analysis for the UV curable quantum dot nanocomposites
Source: Data Brief. 2016 Jan 13;6:614–8. doi: 10.1016/j.dib.2016.01.006 (PMC4735468; doi:10.1016/j.dib.2016.01.006)
Supplement: Supplementary file 1 — Supplementary material [file mmc1.zip › Supplementary Tab1.docx]

**Tab.1** The **lifetime** data.

| $\lambda\text{em}$ | Before curing | After Curing |
| --- | --- | --- |
| 510 nm  540 nm  620 nm | 23.71ns  24.55ns  23.52ns | 1.29ns  2.74ns  2.45ns |
