# Supplementary figures and images for: Quantum yield and lifetime data analysis for the UV curable quantum dot nanocomposites
Source: Data Brief. 2016 Jan 13;6:614–8. doi: 10.1016/j.dib.2016.01.006 (PMC4735468; doi:10.1016/j.dib.2016.01.006)

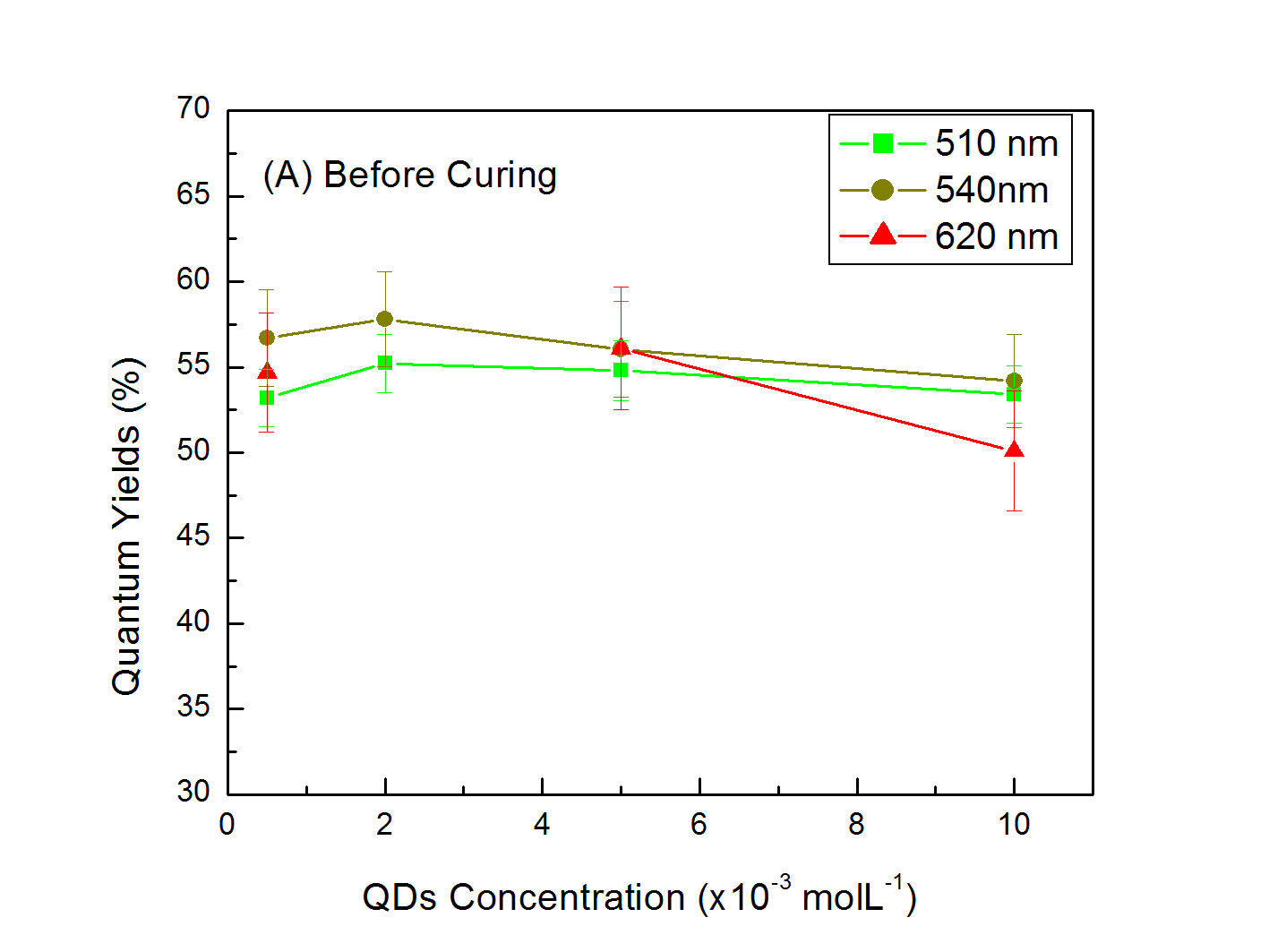

Supplement: Supplementary file 1 — Supplementary material [file mmc1.zip › Supplementary Fig 1A.JPG]

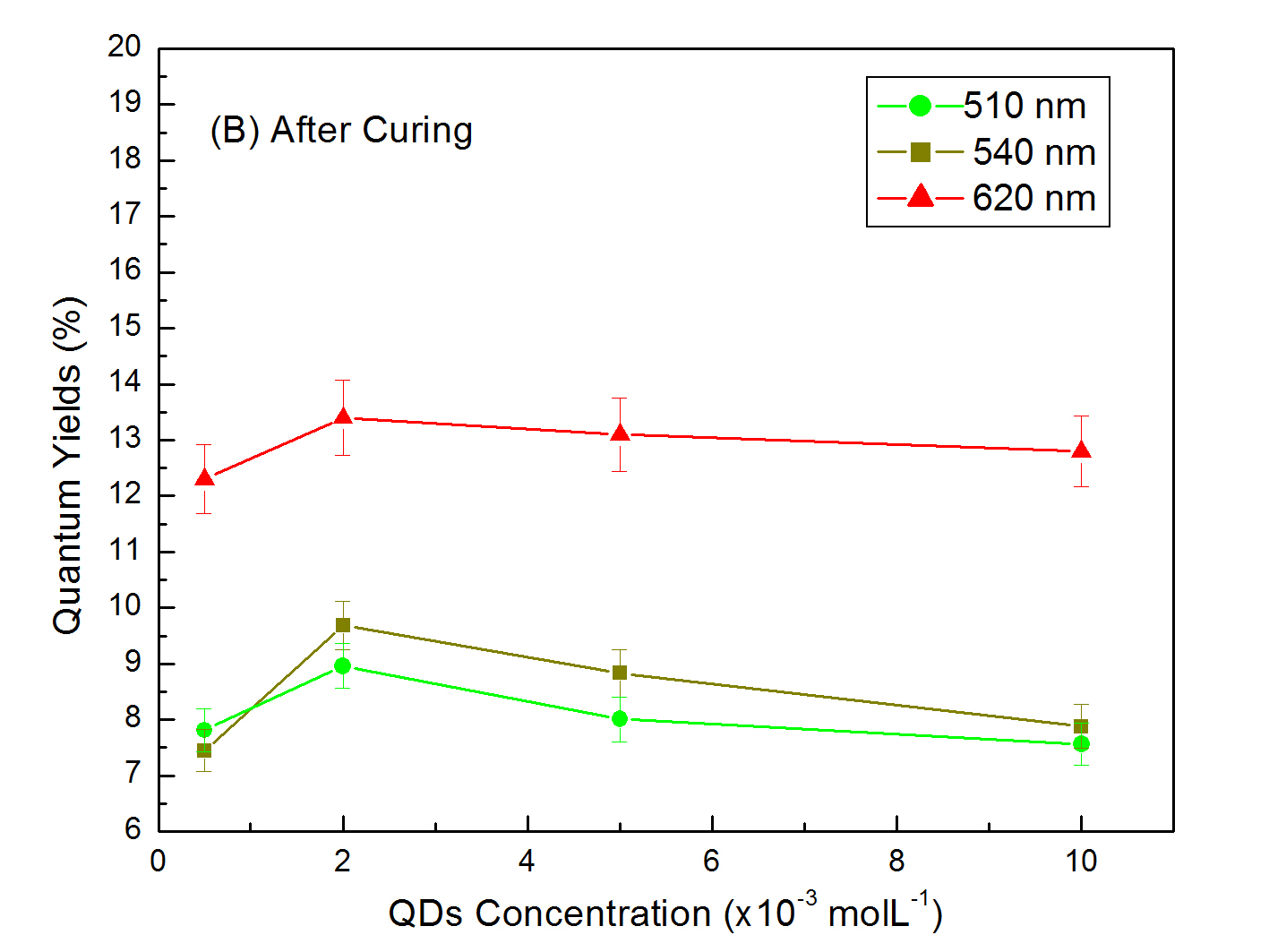

Supplement: Supplementary file 1 — Supplementary material [file mmc1.zip › Supplementary Fig1 B.JPG]
