# Supplementary material for: Quantum yield and lifetime data analysis for the UV curable quantum dot nanocomposites
Source: Data Brief. 2016 Jan 13;6:614–8. doi: 10.1016/j.dib.2016.01.006 (PMC4735468; doi:10.1016/j.dib.2016.01.006)
Supplement: Supplementary file 2 — Supplementary material [file mmc2.docx]

**Conflict of Interests**

The authors declare that there is no conflict of interests regarding the publication of this paper.
